# Patients with IgG1-anti-red blood cell autoantibodies show aberrant Fc-glycosylation

**DOI:** 10.1038/s41598-017-08654-y

**Published:** 2017-08-15

**Authors:** Myrthe E. Sonneveld, Masja de Haas, Carolien Koeleman, Noortje de Haan, Sacha S. Zeerleder, Peter C. Ligthart, Manfred Wuhrer, C. Ellen van der Schoot, Gestur Vidarsson

**Affiliations:** 1 Department of Experimental Immunohematology, Sanquin Research, Amsterdam, and Landsteiner Laboratory, Academic Medical Centre, University of Amsterdam, Amsterdam, The Netherlands; 2 Erythrocyte Serology, Sanquin, Amsterdam, The Netherlands; 3 Center for Proteomics and Metabolomics, Leiden University Medical Center, Leiden, The Netherlands; 4 Department of Immunopathology, Sanquin Research and Landsteiner Laboratory Academic Medical Center, University of Amsterdam, Amsterdam, The Netherlands; 5 Department of Hematology, Academic Medical Center, University of Amsterdam, Amsterdam, The Netherlands

## Abstract

Autoimmune hemolytic anemia (AIHA) is a potentially severe disease in which red blood cells (RBC) are destroyed by IgG anti-RBC autoantibodies which can lead to hemolysis. We recently found IgG Fc-glycosylation towards platelet and RBC alloantigens to be skewed towards decreased fucosylation, increased galactosylation and sialylation. The lowered core-fucosylation increases the affinity of the pathogenic alloantibodies to FcγRIIIa/b, and hence RBC destruction. It is known that in autoimmune diseases plasma IgG1 galactosylation and sialylation are lowered, but Fc-glycosylation of RBC-specific autoantibodies has never been thoroughly analyzed. We investigated by mass spectrometry the N-linked RBC autoantibody and plasma IgG1 Fc-glycosylation in relation to occurrence of hemolysis for 103 patients with a positive direct antiglobulin test (DAT). We observed that total IgG1 purified from plasma of patients with RBC-bound antibodies showed significantly decreased galactosylation and sialylation levels compared to healthy controls, similar to what previously has been shown for other autoimmune diseases. The anti-RBC- autoantibodies showed a profile with even lower galactosylation, but higher sialylation and lower bisection levels. In contrast to alloantibodies against RBCs, RBC-bound IgG1 Fc-fucosylation was not different between healthy controls and patients. Analysis of anti-RBC Fc-glycoprofiles suggested that lower bisection and higher galactosylation associate with lower Hb levels.

## Introduction

Autoimmune hemolytic anemia (AIHA) is a potentially severe disease in which red blood cells (RBC) are destroyed by the action of autoantibodies. Most clinically relevant RBC autoantibodies of IgG class are so called warm autoantibodies binding at 37 °C and lead to extravascular hemolysis by destruction of the opsonized RBC by the phagocytes in the spleen and liver through IgG-Fc receptors (FcγR)^1^. Binding of immunoglobulin G (IgG) and IgM class RBC autoantibodies may result in activation of the classical pathway complement system. Complement deposition on the RBC membrane may reduce cell survival through either extravascular hemolysis via binding to complement receptor-bearing phagocytes in the spleen and liver or, rarely, to intravascular hemolysis if a membrane attack complex is formed^2^. The severity of hemolysis differs per patient and biomarkers correlating with the rate of RBC destruction are lacking. In routine diagnostics, the direct antiglobulin test (DAT) -also known as Coombs’ test- is performed to detect the presence of RBC autoantibodies or fragments of complement proteins when hemolytic anemia is suspected. The combination of laboratory signs of hemolysis with a positive DAT test are diagnostic for AIHA. However, between 2% and 10% of AIHA patients are DAT negative^3^. Vice versa, a positive DAT in the absence of hemolysis is found in 7–8% of all hospitalized patients, indicating that the test does not always has the desired specificity. A positive DAT is often found in patients or healthy blood donors that show no signs of hemolysis^4^.

IgG, existing as four subclasses (IgG1–4), is the most abundant immunoglobulin isotype in the human plasma and is well known for its capacity to recognize pathogens and to evoke strong humoral and cellular effector functions. IgG1 is by far the most abundant subclass, and is the primary antibody formed against T-cell dependent protein antigens^5^. IgG consists of two heavy and two light chains and is divided in the fragment antigen binding (Fab) part and fragment crystallizable (Fc) part, based on the functional activity. While the Fab part provides its functional activity by recognizing the antigen, the Fc part mediates binding to FcγR. The composition of the N-linked sugar moiety (glycan) attached to the Fc region at position 297 of the IgG-Fc tail influences the binding affinity to IgG Fc receptors (FcγR) on effector cells^6–8^. In addition to binding of C1q, activation of the complement cascade can also be modulated by Fc-linked glycans^5^, and some experimental although inconclusive evidence to support this, has been published^9–12^. The N297 glycan consists of an invariant core structure containing two N-acetylglucosamines (GlcNAc) and three mannoses. On top of this structure, galactoses, sialic acids, bisecting GlcNAc (bisection) or core-fucose can be attached^6^. Although the level of fucosylation and bisection of total-IgG is rather stable in a given individual, the level of IgG galactosylation and sialylation is significantly reduced with increasing age^13^. This lowered mean level of galactosylation of IgG1 circulating in plasma is also observed in patients with some autoimmune and infectious diseases^14^. During pregnancy, the mean level of IgG1-galactosylation increases, which is associated with reversal of rheumatoid arthritis during pregnancy^15^. We hypothesize that the type of glycoforms of antibodies involved in immune-mediate blood cell destruction are correlated with disease severity.

In earlier work, we analyzed the allo-immune responses in pregnancy and determined the glycoforms of antigen-specific IgG1^16–19^. Compared to the glycoforms of total IgG detected in plasma of these women, antigen-specific anti-D, anti-K and anti-Human Platelet Antigen 1a were found to be skewed towards low fucosylation^16, 18, 19^, a feature that has only been described for anti-HIV and anti-dengue antibodies^20, 21^, but never for any other immune response. This lowered core-fucosylation is known to increase the affinity to FcγRIIIa and FcγRIIIb, which correlated with the level of cytopenia in affected neonates^16, 18, 19^. Furthermore, we observed an additional role for an increased level of galactosylation in predicting severe clinical outcome of Hemolytic Disease of the Fetus and Newborn.

Here, we investigated the glycoprofile of autoantibodies directed against RBC and evaluated whether the glycosylation pattern is related to detection of complement deposition on the RBC membrane and hemoglobin (Hb) level in IgG-mediated AIHA.

## Patients and Methods

### Patient samples

114 patients with a positive DAT for IgG were tested. Their blood was sent to the Department of Immunohematology Diagnostic Services, Sanquin, Amsterdam, The Netherlands, to elucidate the cause of hemolysis or for alloantibody identification in pregnancy or prior to transfusion (e.g. before surgery). Samples were included when leftover material was available and used according to the Dutch established codes of conduct for responsible use and approved by our institute^22^. The material was used anonymously except for age and sex. These parameters were used for matching cohorts for comparisons whenever possible, e.g. between healthy controls and patients. Patients with anti-RBC autoimmune reactions tend to be of older age, as was the case in this study, but exceptions were noted. Due to the limited size of the patient cohort, a full matching for both age and sex was not possible for comparisons between patient subgroups. Hemolysis parameters (Hb, haptoglobin, bilirubin and lactate dehydrogenase (LDH) levels and number of reticulocytes) were provided by the referring center as part of the diagnostic work-up to judge the clinical relevance of the laboratory test results.

### Serological analysis of patient samples

Autoantibodies were detected by using the DAT. Samples were included if the DAT performed with anti-IgG was positive and IgG antibodies were detected in the eluate prepared from the red cells. The DAT was performed by column technique (DC-screening I, BioRad, Veenendaal, The Netherlands) according to the instructions using 0.8% unwashed RBCs and by tube testing with monospecific anti-human IgG and anti-C3d (Sanquin Reagents, Amsterdam, the Netherlands) using 3% RBCs (three times washed with cold PBS). Eluates were prepared with Pelistrip elution kit (Sanquin Reagents) and tested with the indirect antiglobulin test (IAT) with the addition of polyethylene glycol (PEG). Only if the eluate tested positive with all test cells (n ≥ 8) the presence of anti-RBC-reactive autoantibodies could be reported.

Samples were categorized into three groups; 1. hemolysis (based on a low Hb (≤11.3 g/dL) and haptoglobin (≤0.3 mg/L) level and increased LDH (>225 U/L), reticulocyte count (>25/1000 erythrocytes) and/or bilirubin level (≥17 mmol/L), 2. hemolysis unlikely or absent (“no hemolysis”) (low or normal Hb and normal haptoglobin, LDH, reticulocyte count or bilirubin level), or 3. hemolysis inconclusive (‘’hemolysis unknown”: inconclusive or unknown laboratory results).

### Purification of anti-RBC antibodies

RBC autoantibodies were isolated from 500 µl freshly obtained RBCs after washing 6 times with 9 ml cold PBS (phosphate buffered saline). 500 µl acid (pH 2.3) elution buffer (Sanquin reagents) was added to the packed cells followed by direct centrifugation (2300 g, 90 sec.) after which the supernatant was added to 25 µl neutralization buffer (214 mM Tris, 22 mM Na_2_HPO_4_). To remove the RBC debris an extra centrifugation step (2300 g, 1 min.) was performed. RBC from healthy blood donors were used as negative controls.

The amount of IgG1 present in the eluate was measured by ELISA (NUNC-Immuno, Maxisorp, Sigma Aldrich, St Louis, MO). The plate was coated with mouse-anti-human IgG1 (clone M1325, Sanquin Reagents) and mouse-anti-human IgG-Fc-HRP-labeled: Southern Biotech (9040–05) for detection. Fully human recombinant antibodies (IgG1-kappa GDOB1) was used as a standard to calculate the IgG1 concentrations^23, 24^.

### IgG purification from plasmas and eluates

IgG1 present in plasma (“total”) and RBC-bound-IgG1 antibodies were purified using protein G Sepharose 4 Fast Flow beads (GE Healthcare Life Sciences) in a 96-well filter plate (Orochem, Naperville, IL). Eluates (300 µl) and plasmas (2 µl) diluted in 150 µl PBS were incubated for one hour at room temperature, shaking 450 RPM (Heidolph Titramax 100, Namur, Belgium) with 15 µl protein G Sepharose beads, using empty wells (protein G without sera) as negative control. The plates were washed six times: three times with 300 µl PBS and three times with water. The bound antibodies were then eluted using 100 µl 100 mM formic acid, as described previously^25^. No IgG was found by mass spectrometry in eluates from blank wells or prepared from RBCs from healthy blood donors, proving there was only anti-RBC present in the eluates.

Eluates were transferred into V-bottom plates before drying by vacuum centrifugation for 2.5 hours and then dissolved in 20 µl 50 mM ammonium bicarbonate followed by 10 minutes of shaking. To optimize the proteolytic cleavage of the eluates by trypsin, 1 mg RapiGest (RapiGest SF surfactant (Waters, Etten-leur, the Netherlands) was dissolved in 50 µl 50 mM ammonium bicarbonate. 2 µl of the RapiGest solution was added to the eluates and incubated at 100 °C for five minutes.

Both the RBC and total IgG fractions from the same patient were then subjected to proteolytic cleavage by trypsin (Promega, Leiden, the Netherlands). 20 µg trypsin was diluted in 50 µl ice cold 20 mM acetic acid and 1300 µl ice cold water. 20 µl of this trypsin dilution (300 ng trypsin) was added to each well directly, followed by 10 minutes of shaking. Samples were incubated overnight at 37 °C. To get rid of the RapiGest, 0.5% TFA was added to the eluates followed by incubation for 30 minutes at 37 °C and centrifugation at 15817 × g for ten minutes. Samples were stored at −20 °C until measurement.

### Mass spectrometric IgG Fc glycosylation analysis

Nano liquid chromatography-tandem mass spectrometry (LC-MS/MS) was performed largely as we described previously^18, 19^, see supplementary methods. In the current study we focused on IgG1, without analyzing IgG3 due to its possible interference with IgG2 at the glycopeptide level^26^. Primary data processing, including calibration, alignment, and peak extraction, was performed as described previously, using Bruker DataAnalysis 4.0, msalign2 and the in-house developed tool 3D Max Extractor, respectively^27, 28^, see supplementary methods. Mass spectrometric glycosylation profiles were included for analysis when the sum of the signal intensities of all glycopeptide species (Supplementary Table 1) divided by the background of the same spectrum was above 100. The total level of glycan traits was calculated as described in Supplementary Table 2.

### Statistical analysis

Statistical analyzes were performed using GraphPad Prism version 6.00 for Windows (GraphPad Software, La Jolla, CA) and IBM SPSS Statistics for Windows version 21.0 (IBM Corp, Armonk, NY). As the study population (glycosylation traits and Hb levels) showed normal distribution according to the Shapiro-Wilk normality test, statistical analysis was performed using parametric tests. For the comparison between anti-RBC IgG1 Fc-glycosylation and total IgG1 Fc-glycosylation, two-tailed paired t-tests were performed for the four derived glycosylation traits galactosylation, sialylation, bisection and fucosylation. The correlation between hemolysis severity, as measured by hemoglobin levels, and anti-RBC IgG1 Fc-glycosylation traits was assessed by the Pearson correlation test. This was done for the patient cohort in its entirety, and also for the subsets “patients with hemolysis”, “patients without hemolysis” and “patients with unknown hemolysis status”. In addition, the patients were compared to the healthy controls by three two-tailed, unpaired t-tests per glycosylation trait: healthy vs. all patients, healthy vs. patients with hemolysis and healthy vs. patients without hemolysis. The effect of IgG1 Fc-glycosylation on complement activation and C3d binding to RBCs was assessed by two-tailed, unpaired t-tests between C3d+ and C3d− patients for the derived glycosylation traits on total IgG1 and anti-RBC-IgG1. In total, this resulted in 48 individual statistical tests, for which the significance threshold (α) was corrected by the Benjamini-Hochberg procedure with a false discovery rate (FDR) of 5%, resulting in α = 0.0074 throughout the study.

## Results

### Skewed anti-RBC Fc-glycosylation in autoantibodies

**Figure 1:**
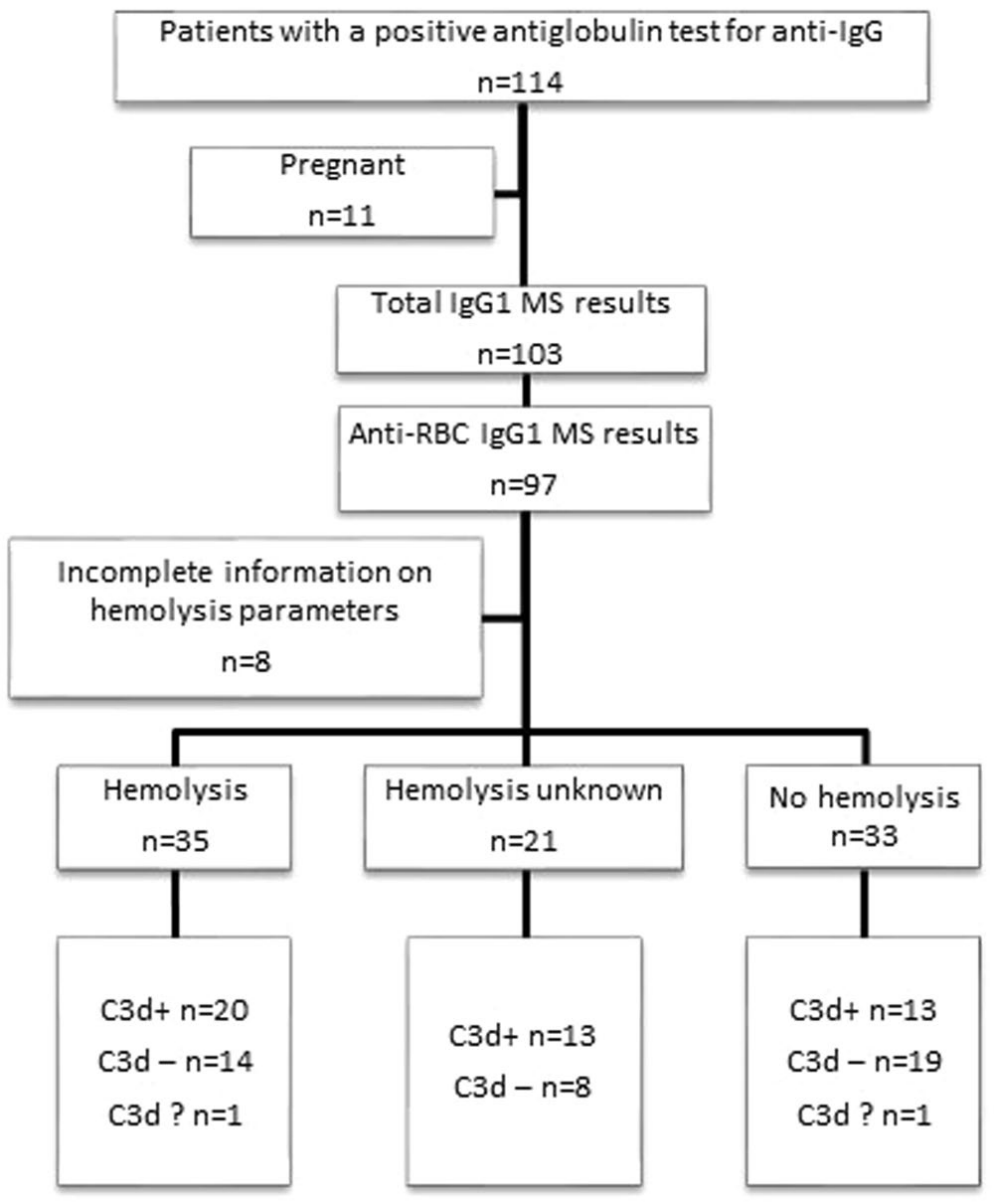
Flowchart of patients with autoantibodies against RBCs included in the study. Mass spectrometric (MS) results of IgGs were excluded when signals were below the intensity threshold. Samples of 11 pregnant women were excluded because it is known that pregnancy effects the Fc-glycoyslation^37^. Samples were categorized into three groups; 1. Hemolysis (Hb ≤ 11.3 g/dL, haptoglobin ≤0.3 mg/L, LDH > 225 U/L, reticulocyte count >25/1000 erythrocytes and/or bilirubin level ≥17 mmol/L, 2. No hemolysis (Hb > 11.3 g/dL, haptoglobin 0.3–2.0 mg/L, LDH 135–225 U/L, reticulocyte count 5–25/1000 erythrocytes or bilirubin level known and <17 mmol/L), or 3. Hemolysis unknown: Hb ≤ 11.3 g/dL and inconclusive or unknown other laboratory results.

**Figure 2:**
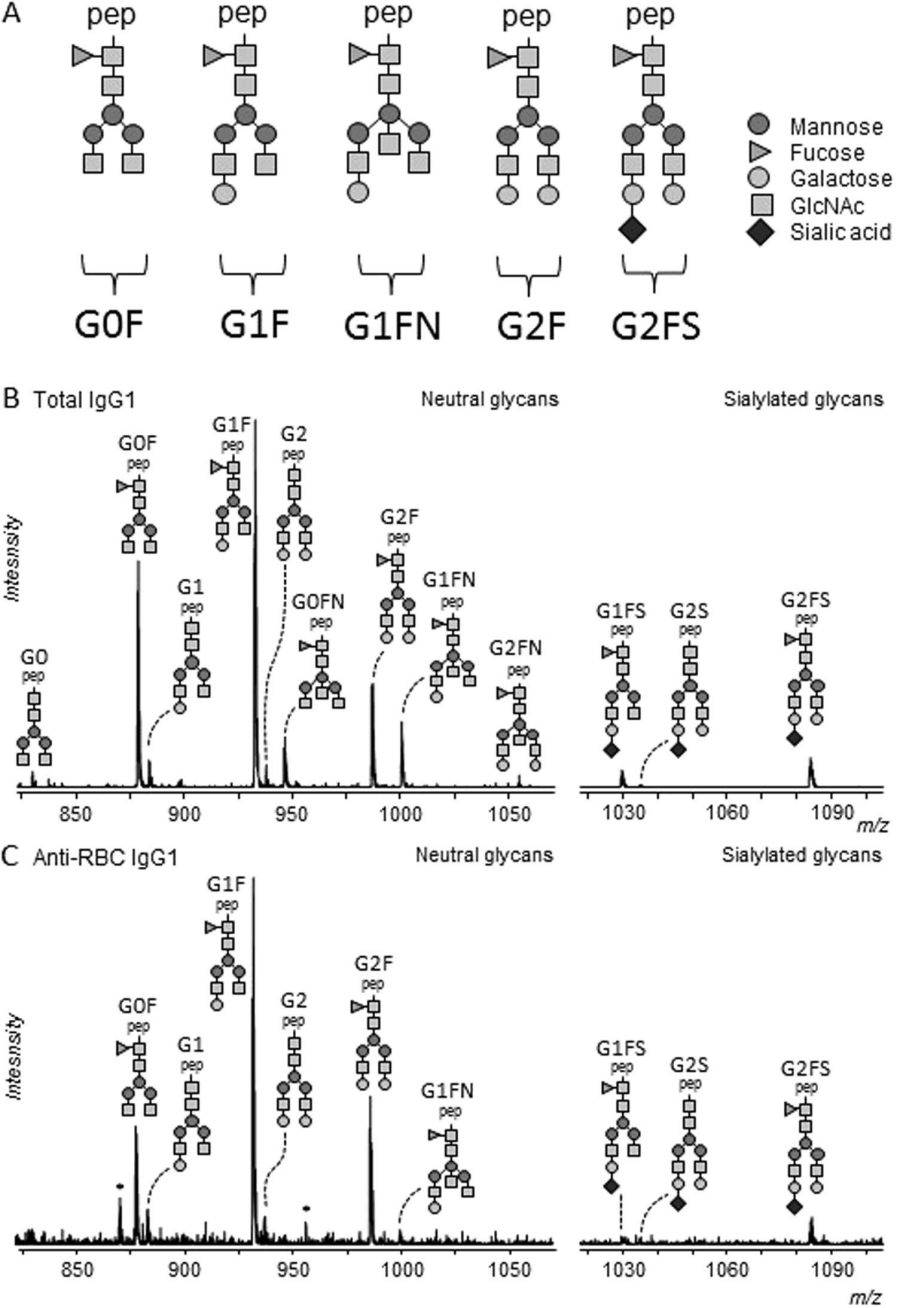
Examples of IgG Fc N-glycan structures. On top of the core structure, galactoses (G), bisecting GlcNAc (N), sialic acids (S) or fucose (F) can be attached: Triangle, fucose; darker circle, mannose; lighter circle, galactose; diamond, N-acetylneuraminic (sialic) acid (**A**). Mass spectrometric analysis of Fc glycopeptides encompassing N297 of total IgG1 (**B**) and anti-RBC IgG1 (**C**). Spectra from a representative patient are shown, with mass/charge ratio (m/z) of the glycopeptides on the x-axis and relative abundance on the y-axis. Asterisks indicate non-glycopeptide signals. Triple-protonated glycopeptide signals are shown in this figure.

To analyze RBC specific glycosylation patterns, specific RBC antibodies were isolated by making eluates of RBC followed by glycosylation analysis of Fc-glycopeptides using mass spectrometry. Anti-RBC IgG1 Fc-glycosylation profiles were obtained from 97 out of 114 patients (Fig. 1) with RBC autoantibodies. The most prevalent N-linked glycan structures were found to be highly heterogeneous in number of galactose residues with the most prevalent glycoforms carrying no (G0F), one (G1F and G1FN) or two galactose residues (G2F and G2FS) (Fig. 2).

**Figure 3:**
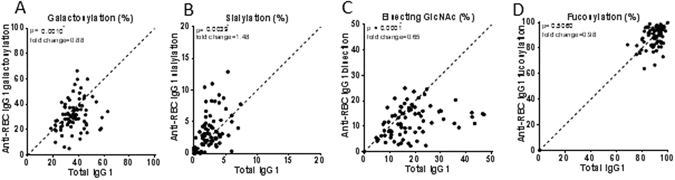
Auto-antibodies against RBCs show skewing of anti-RBC IgG1 galactosylation, bisection and sialylation. Total- (x-axis) versus antibody specific (y-axis) IgG1 shows a decreased anti-RBC (**A**) galactosylation and (**C**) bisecting GlcNAc, increased (**B**) sialylation, and no skewing of (**D**) fucosylation. Statistical analysis was done using a paired t-test, after 5% FDR correction *p*-values ≤ 0.0074 were considered statistically significant. The fold change indicates the fold difference between the average of the antigen specific IgG1 glycosylation feature, divided by the average of the total IgG1 glycosylation feature. Asterisks indicate significant *p*-values after correction for multiple comparisons.

The Fc-glycoprofiles of purified anti-RBC specific IgG were compared in a pairwise manner with that of plasma-derived total IgG with respect to galactosylation, sialylation, bisection and fucosylation (Fig. 3). For the group as a whole, IgG1 Fc-galactosylation and bisecting GlcNAc were lowered and sialylation was increased for the anti-RBC-IgG1 autoantibodies compared to total IgG1 (Fig. 3A–C). Importantly, no significant change was observed for Fc-fucosylation in anti-RBC specific IgG1 compared to total IgG1 (Fig. 3D).

### Anti-RBC specific IgG bisection and galactosylation correlate with severity of hemolysis

**Figure 4:**
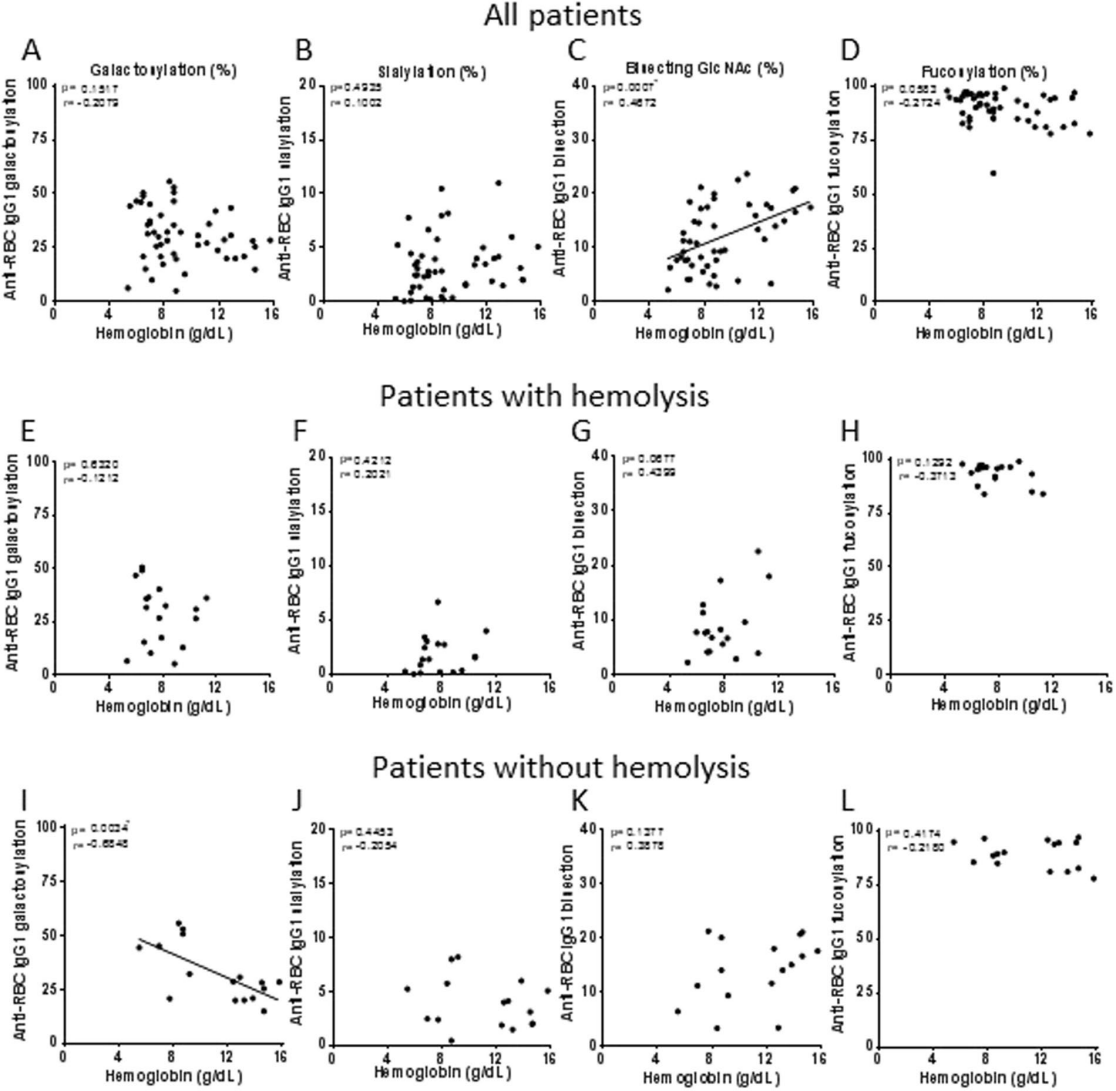
Low anti-RBC specific galactosylation and bisecting GlcNAc correlate with severe anemia in patients with RBC autoantibodies. Anti-RBC IgG1 galactosylation, sialylation, bisecting GlcNAc, fucosylation (y-axis) and hemoglobin level (x-axis) for IgG1 is shown for (**A**–**D**) all patients with RBC autoantibodies, (**E–H**) patients with hemolysis and (**I**–**L**) patients without hemolysis. Bisecting GlcNAc shows a correlation with Hb level including all patients RBC autoantibodies (**C**) and galactosylation shows a negative correlation with Hb level in patients without hemolysis (**E**). Statistical analysis was done using the Pearson correlation test, after 5% FDR correction *p*-values ≤ 0.0074 were considered statistically significant Asterisks indicate significant *p*-values after correction for multiple comparisons.

To investigate whether Fc-glycosylation has an effect on clinical outcome, glycosylation profiles were correlated to hemoglobin levels (Fig. 4). Of the 97 patients for which we succeeded in obtaining a reliable anti-RBC Fc-glycosylation profile, clinical data were available for 89 patients. Of these 89 analyzed patients (Fig. 1), the hemoglobin level was only known in 49 cases. The Hb for all patients (n = 49) showed an average of 9.2 g/dL (5.3–15.8 g/dL) with as expected hemolytic patients (n = 18) having a lower average Hb of 7.7 g/dL (5.3–11.3 g/dL) compared to 11.3 g/dL in non-hemolytic patients (5.5–15.8 g/dL) (n = 16). For the group as a whole, we found no correlation for the glycosylation traits with Hb, except for a positive correlation with anti-RBC-IgG1 bisection (p = 0.0007, r = 0.4672) (Fig. 4A–D). As this represents a heterogeneous group, the patients were divided based on the presence of hemolysis as depicted in Fig. 1, and the subgroups were separately analyzed (patients with hemolysis (Fig. 4E–H), with no hemolysis (Fig. 4I–L) and with unknown hemolysis (Supplementary Fig. 1A–D)). No significant associations were observed, except in the group of patients with no hemolysis we found anti-RBC IgG Fc-galactosylation to correlate negative with hemoglobin level (p = 0.0034, r = −0.685).

### Low IgG1 Fc galactosylation and sialylation in patients with RBC-autoantibodies

**Figure 5:**
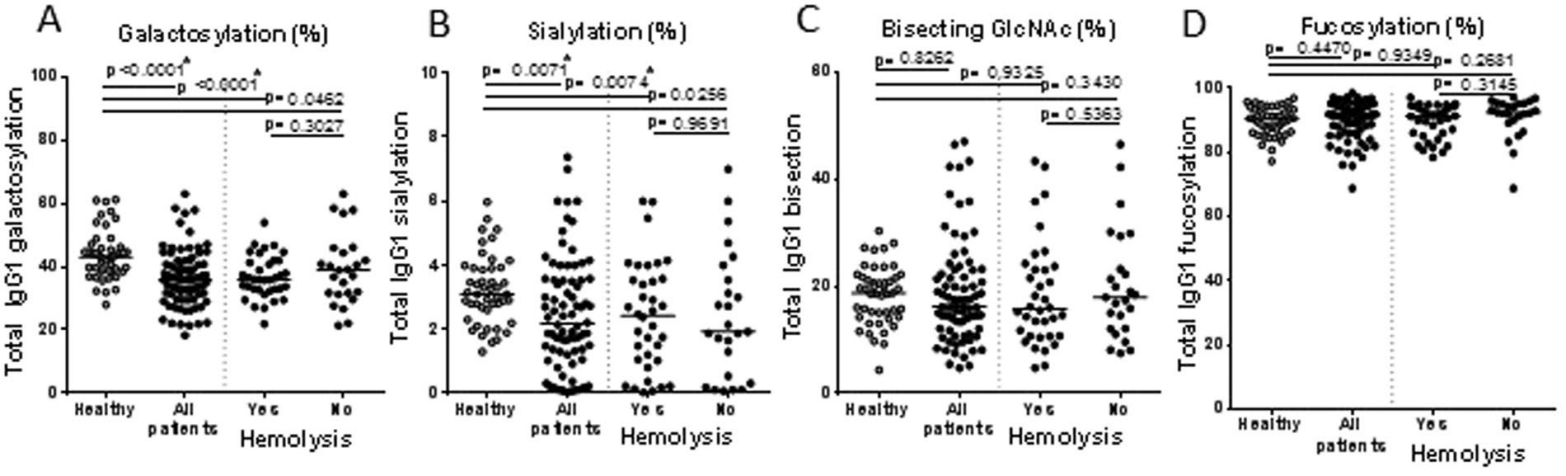
Lack of IgG1 galactosylation and sialylation in patients with autoantibodies compared to healthy controls. Total IgG1 glycosylation (y-axis) in patients with autoantibodies against RBCs (black dots) is different compared to healthy controls (grey dots) (x-axis). Fc-galactosylation (**A**) and sialylation (**B**) are lower in patients with RBC autoantibodies. Fc-bisecting GlcNAc (**C**) and fucosylation (**D**) are comparable in both groups. No difference was found between patients with and without hemolysis for total galactosylation (**A**), sialylation (**B**), bisecting GlcNAc (**C**) and fucosylation (**D**). The data present individual observations with median. Statistical analysis was done using an unpaired t-test between the healthy controls and all patients, patients with hemolysis and patients without hemolysis, after 5% FDR correction *p*-values ≤ 0.0074 were considered statistically significant. Asterisks indicate significant *p*-values after correction for multiple comparisons.

To investigate the Fc glycopeptides of IgG1 isolated from plasma of patients with RBC autoantibodies, we analyzed the plasma IgG1 of 103 patients with a positive DAT result (Fig. 1) and compared them with Fc-glycopeptides of healthy controls (n = 47). Patients and controls were matched for age and sex (Supplementary Tables 3 and 4). We observed that IgG1 purified from plasma of these patients shows decreased galactosylation (average 37%) and sialylation (2.4%) levels compared to healthy controls (47% and 3.3%, respectively; p < 0.0001 and p = 0.0015) (Fig. 5A-B). The decreased galactosylation of IgG1 in patients was only seen in the group of patients with hemolysis and not in the other subgroups (Fig. 5A). The levels of bisecting GlcNAc or fucosylation on Fc-IgG1 in patients with RBC autoantibodies did not show a difference compared to healthy controls (Fig. 5C,D).

### IgG1 Fc-glycosylation does not discriminate between C3-opsonized and unopsonized RBC with autoantibodies

To assess whether complement activation and C3d binding to RBCs is affected by IgG1 Fc-glycosylation we compared total and specific IgG1 galactosylation, sialylation, bisection and fucosylation in patients with a positive or negative test results for anti-C3d. No difference in total- or specific IgG1 glycosylation pattern was found between C3d positive and C3d negative RBC isolated from patients (Supplementary Fig. 2).

## Discussion

In this study, we analyzed the IgG1 Fc-glycosylation profiles for RBC autoantibodies of 97 patients and found a significantly different profile of glycoforms compared to that of plasma derived glycoforms, with lower levels of galactosylation and bisection, and higher levels of sialylation. But unlike previously reported for alloimmune anti-platelet and RBC antibodies, no marked changes were found for core fucosylation suggesting a different origin in these immune responses. We found that both lowered anti-RBC bisection for all patients and increased galactosylation in patients with no hemolysis correlated with more severe anemia. In general, we also found a remarkable decrease in galactosylation and sialylation of plasma derived IgG1 in patients with RBC autoantibodies compared to age matched healthy controls, which is in line with observations reported for other autoimmune diseases^14, 29^.

In alloantibodies against RBCs in pregnant women we previously found that IgG1 Fc-glycosylation profiles of anti-D, -c, -E and -K differed markedly from total IgG-glycosylation but also from each other, especially for Fc-fucosylation^30^. IgG-Fc afucosylation is known for its strong ability to increase FcγRIIIa/b affinity, leading to enhanced cytotoxicity^14^. In allo anti-K and anti-RhD, but also in anti-HPA-1a IgG1 antibodies, a decreased antigen specific IgG1 fucosylation was found, correlating with more severe disease^16, 18, 30^. In the current study analyzing RBC autoantibodies we do not find a skewing of anti-RBC specific fucosylation, indicating that the driving mechanism underlying alloantibody formation, is different compared to that of autoantibody formation. The only alloantibody in which we did not observe lowered fucosylation was anti-E, an antibody which is often a naturally occurring antibody, not induced by allogeneic RBCs^31^.

In this study we found a positive correlation between anti-RBC IgG1 bisection and Hb level, similar to previous associations for allo-anti-K and Hb levels in newborns^30^. However, very limited, if any, information exists on the functional consequences of the bisecting GlcNAc^32, 33^, and this association was lost when subgroups with or without hemolysis were analyzed separately.

Anti-RBC antibodies have the capacity to elicit RBC clearance via Fc-receptor-mediated destruction but also via complement activation^4^. We found that total IgG1 Fc-galactosylation was lowered in patients with anti-RBC autoantibodies, and RBC-specific autoantibodies were found to have even lower Fc-galactosylation compared to total IgG1. In general, no significant correlation was found between Hb and anti-RBC galactosylation. Only a significant correlation was found in the group with no hemolysis, where a higher galactosylation level of the RBC specific autoantibodies seemed to predict lower Hb. Importantly, increased galactosylation of specific IgG correlating with worse clinical outcome has been described for anti-K, anti-Rhc and anti-HPA-1a alloantibodies^19, 30, 34^. This may be explained by galactosylation increasing the binding affinity to FcγRIIIa^7, 35^, but may also be an effect of increased C1q-binding and further complement-mediated deposition and/or lysis^10^ (Dekkers *et al*., submitted for publication). The reason why no association with galactosylation level of these specific antibodies was found in the total group and in patients with hemolysis may be due to enhanced catabolic degradation of the most potent antibodies. This may explain why it is only found in patients without clear signs of hemolysis, however studies in animal models of antibody-mediated anemia will be required to find out if enhanced clearance of more clinically active glycoforms of antibodies occurs.

At least two previous groups have studied total and specific galactosylation in patients with AIHA and also found no clear correlation with clinical outcome^34, 36^. However in these studies, different techniques were used that did not discriminate between Fc-linked and Fab-linked glycans, making a direct comparison with our study impossible. Recent research showed that sialylation negatively affects C1q binding to IgG1 and downstream activation^10^. It has also been suggested that a lack of galactosylation results in an interaction of mannose-binding lectin with N-acetylglucosamines and thereby leads to more efficient complement activation^11^. In this study, no signs of such associations were found.

Although the exact mechanism driving the immune system to modify the glycosylation of IgGs remains unclear, Fc-glycosylation is known to be affected by age, sex and pregnancy and a variety of chronic autoimmune diseases^13, 15, 29, 37^. Since the healthy cohort was matched for age and sex, and all pregnant women were excluded, our findings cannot be explained by these possible cofounders. IgG galactosylation in the patient cohort is generally lower compared to controls, and as expected gradually decreases with increasing age (Supplementary Fig. 3). Low total IgG galactosylation and sometimes also sialylation levels are described in several inflammatory diseases such as rheumatoid arthritis, systemic lupus erythematosus and Crohn’s disease, indicating that IgG Fc-agalactosylation is a biomarker for immune activation^14, 29, 38^. Whether this is a causative agent for the induction of autoimmune reaction or a consequence of a chronic immune activation is unknown. As agalactosylated IgG has lowered affinity for FcγRIIIa/b and C1q, high levels of galactosylated total IgG may raise the threshold for immune activation. Hence, low galactosylation of total IgG may theoretically aggravate the disease. This notion is supported by the observation that in rheumatoid arthritis a lowered total IgG-galactosylation seems to worsen clinical symptoms^29^. In this study, a similar decrease of total IgG galactosylation and sialylation was found in patients with anti-RBC autoantibodies, compared to healthy controls, and particularly in those with hemolysis. A possible limitation of this study is that presumably some of the included patients might suffer from AIHA secondary to an – not yet diagnosed – autoimmune disease. AIHA can occur without an underlying disease (primary or idiopathic AIHA) but approximately 50% is associated with an underlying disease (secondary AIHA)^39, 40^. Also, due to the size of the patient cohort, we were not able to analyze an association of male and female anemia in the light of differential antibody properties. This may affect the outcome as the sexes differ slightly in basal Hb level. This gender difference is however smaller than the observed difference in anemia and is therefore not expected to have a big impact on our results.

Other limitation of this study is that both antigen specificity and antigen density are unknown. But also the fact that we only investigated Fc-glycosylation patterns of IgG1, as we were unable to analyze the other subclasses due to their interferences at the glycopeptide level^26^, is a limitation. In warm autoantibodies, the RBC survival is influenced by IgG subclass, with IgG1 being the most commonly encountered subclass, and IgG3 decreases RBC life span, followed by IgG2 and IgG4^39^. In the present cohort, 29 patients were tested for the presence of IgG2, IgG3 and IgG4 in addition to IgG1 because of a strong positive DAT result (n = 29). Of those, 11 patients were tested positive for IgG2, 14 patients were tested positive for IgG3 and five patients were also positive for IgG4, but no quantitative data on the relative levels of those subclasses in these patient was available^26^. But since we have found that the glycosylation profiles for IgG1 and IgG3 are strongly correlated (Sonneveld *et al*. manuscript in preparation), we do not consider this as a major limitation of our study.

In conclusion, it is clear from this study that autoantibodies against red cells have completely different glycosylation response as seen for alloantibodies. This is most striking for fucosylation, as lowering of core-fucosylation is apparently a unique feature of alloantibodies^30^, but never for autoantibodies. Total IgG1 Fc-galactosylation and sialylation were found to be lowered in patients with autoantibodies against RBCs compared to healthy controls, similar as has been described for several other autoimmune diseases.

## Electronic supplementary material

Supplementary Information
