# Supplementary material for: Patients with IgG1-anti-red blood cell autoantibodies show aberrant Fc-glycosylation
Source: Sci Rep. 2017 Aug 15;7:8187. doi: 10.1038/s41598-017-08654-y (PMC5557851; doi:10.1038/s41598-017-08654-y)
Supplement: Supplementary file 1 — Supplementary Information [file 41598_2017_8654_MOESM1_ESM.pdf]

Patients with IgG1-anti-red blood cell autoantibodies show aberrant Fc-glycosylation

-Supplementary information -

Myrthe E. Sonneveld[1], Masja de Haas[2], Carolien A.M. Koeleman[3], Noortje de Haan[3], Sacha S. Zeerleder[4,5], Peter C. Ligthart[2], Manfred Wuhrer[3], C. Ellen van der Schoot[1], Gestur Vidarsson[1*].

1 Department of Experimental Immunohematology, Sanquin Research, Amsterdam, and Landsteiner Laboratory, Academic Medical Centre, University of Amsterdam, Amsterdam, The Netherlands; 2 Erythrocyte Serology, Sanquin, Amsterdam, The Netherlands; 3 Center for Proteomics and Metabolomics, Leiden University Medical Center, Leiden, The Netherlands; 4 Department of Immunopathology, Sanquin Research and Landsteiner Laboratory Academic Medical Center, University of Amsterdam, Amsterdam, The Netherlands; 5 Department of Hematology, Academic Medical Center, University of Amsterdam, The Netherlands.

Sonneveld et al, supplementary methods

Supporting info:

Mass spectrometric analysis of glycopeptides was performed as described previously by *Kapur et al. 2014*. Aliquots of the tryptic digests of total IgG (200 nl) and anti-RBC antibodies (5µl) were applied to a C18 PepMapTM 100um x 20mm trapping column (5 µm, 100 Å; Thermo Scientific) and washed with 100% A (0.1% formic acid in water) at 15 µl/min for 2 minutes. Following valve switching, tryptic (glyco-)peptides were separated on a reverse-phase column (Acclaim C18 PepMap;75 µmx150 mm, 2 µm, 100 Å; Thermo Scientific) at a flow rate of 700 nl/min. The gradient applied was as follows; 18% eluent B (95% acetonitrile, 5% water) in 5 min and 3-27% eluent B in the next 15 min, followed by an isocratic elution with 70% eluent B for 3 min. The LC system was coupled to an AmaZon Speed ETD ion trap MS (Bruker Daltonics, Bremen, Germany) operated in the positive ion mode. The sample was ionized with an CaptiveSprayer (1300 V) using CaptiveSpray tapered spray tip (internal diameter of 20 µm) Microm Bioresources Inc.. The solvent was evaporated at 180 °C with a nitrogen flow of 3 liters/min. A CaptiveSpray nanoBooster (Bruker Daltonics) was mounted onto the mass spectrometer and saturated the nitrogen flow with acetonitrile to enhance the sensitivity (0.2bar). The MS1 ion detection window was set at m/z 550–1800. The HPLC method resulted in separation of the glycopeptides based on the peptide moiety with IgG1 glycopeptides eluting first, followed by the elution of IgG4 and lastly IgG2 and IgG3 glycopeptides (*Wuhrer et al. 2007, Selman et al. 2012, Kapur et al. 2014*). Moreover, glycopeptides with neutral glycan moieties tended to elute earlier than glycopeptides with antennae sialylation, as described before. For both the neutral and the acidic glycopeptides of each IgG subclass, average mass spectra were generated over a 0.6 min elution range using Bruker DataAnalysis 4.0.. Glycopeptides were assigned on the basis of mass, retention time (RT)and CID fragmentation pattern, and interference from Fab glycans as well as from other IgG subclasses and antibodies were excluded. We focused on IgG1, without analyzing IgG3 due to the interference with IgG2. The primary data processing was done as described before (*Selman et al. 2012, Plomp et al. 2015*). First, the mass spectra were calibrated internally with Bruker DataAnalysis 4.0 using a list of known glycopeptides, subsequently the runs were exported to the open mzXML format by Bruker DataAnalysis 4.0 in batch mode. Using msalign2, the runs were aligned to a master-run of a representative sample, using a list of known glycopeptides. For the extraction of the glycopeptide signal intensities, the in-house tool 3D Max Extractor was used. This program examines the data points in a given m/z and RT window, respectively +/- 0.07 Th and +/- 30 s, and reports the maximum intensity observed per predefined analyte (m/z, RT combination). For each IgG1 glycoform the intensities of the first three isotopic peaks, both in their doubly and triply charged form, were background corrected and summed. The absolute values were normalized to the subclass-specific sum of signals. The second isotopic peak for IgG1 G0 and G1S as well as the third isotypic peak for IgG1 G1S were obscured by overlapping peaks, and therefore the signal for these peaks was estimated based on the signal of the remaining isotopic peaks and the theoretical isotopic pattern.

The levels of bisection, fucosylation, and antenna galactosylation and sialylation were calculated on the basis of the normalized intensities of IgG1 Fc glycopeptides according to the formulas presented in supplementary table 2.

Sonneveld et al, supplementary table 1

Supplementary Table 1. Overview of the IgG Fc glycopeptides which were included. The monoisotopic *m/z* value of the 2+ and 3+ charge state are shown, together with the average retention time determined for all N-glycans of each IgG subclass. Glycan compositions are denoted using the following nomenclature: H = hexose, N = N-acetylhexosamine; F = fucose; S = sialic (N-acetylneuraminic acids).

N-glycopeptides	m/z 2+	m/z 3+	retention time (s)
IgG1 H3N4F1S0	1317.526565	878.6868033	336
IgG1 H4N4F1S0	1398.552975	932.70441	336
IgG1 H5N4F1S0	1479.579385	986.7220167	336
IgG1 H3N5F1S0	1419.06625	946.3799267	336
IgG1 H4N5F1S0	1500.09266	1000.397533	336
IgG1 H5N5F1S0	1581.11907	1054.41514	336
IgG1 H3N4F0S0	1244.49761	830.0008333	336
IgG1 H4N4F0S0	1325.52402	884.01844	336
IgG1 H5N4F0S0	1406.55043	938.0360467	336
IgG1 H3N5F0S0	1346.037295	897.6939567	336
IgG1 H4N5F0S0	1427.063705	951.7115633	336
IgG1 H5N5F0S0	1508.090115	1005.72917	336
IgG1 H4N4F1S1	1544.100685	1029.736217	396
IgG1 H5N4F1S1	1625.127095	1083.753823	396
IgG1 H4N5F1S1	1645.64037	1097.42934	396
IgG1 H5N5F1S1	1726.66678	1151.446947	396
IgG1 H4N4F0S1	1471.07173	981.0502467	396
IgG1 H5N4F0S1	1552.09814	1035.067853	396
IgG1 H4N5F0S1	1572.611415	1048.74337	396
IgG1 H5N5F0S1	1653.637825	1102.760977	396
IgG1 H5N4F1S2	1770.674805	1180.78563	396

Sonneveld et al, supplementary table 2

Supplemental Table 2. An overview of the calculations for the derived glycosylation traits.		
Derived trait	Definition	Calculation
Fucosylation	% of *N*-glycans which carry a core fucose	H3N4F1S0 + H4N4F1S0 + H5N4F1S0 + H3N5F1S0 + H4N5F1S0 + H5N5F1S0 + H4N4F1S1 + H5N4F1S1 + H4N5F1S1 + H5N5F1S1 + H5N4F1S2
Galactosylation	% of galactoses per antenna on *N*-glycans	(H5N4F1S0 + H5N5F1S0 + H5N4F0S0 + H5N5F0S0 + H5N4F1S1 + H5N5F1S1 + H5N4F0S1 + H5N5F0S1 + H5N4F1S2) + 0.5 * (H4N4F1S0 + H4N5F1S0 + H4N4F0S0 + H4N5F0S0 + H4N4F1S1 + H4N5F1S1 + H4N4F0S1 + H4N5F0S1)
Sialylation	% of *N*-acetylneuraminic (sialic) acids per antenna on *N*-glycans	H5N4F1S2 + 0.5 * (H4N4F1S1 + H5N4F1S1 + H4N5F1S1 + H5N5F1S1 + H4N4F0S1 + H5N4F0S1 + H4N5F0S1 + H5N5F0S1)
Bisection	% of *N*-glycans which carry a bisecting *N*-acetylglucosamine	H3N5F1S0 + H4N5F1S0 + H5N5F1S0 + H3N5F0S0 + H4N5F0S0 + H5N5F0S0 + H4N5F1S1 + H5N5F1S1 + H4N5F0S1 + H5N5F0S1

Sonneveld et al, supplementary table 3

	Age	Sex	Total fuc	Total gal	Total bis	Total sial
	70	Female	93.15	42.90	15.07	3.48
	69	Female	83.16	28.00	19.51	1.68
	68	Female	77.10	44.33	21.65	3.91
	68	Male	92.23	36.79	27.07	2.94
	66	Male	90.46	39.83	30.32	3.45
	66	Female	85.18	39.42	15.77	2.80
	65	Female	94.70	39.62	4.32	3.30
	65	Male	84.26	40.75	27.20	3.93
	64	Male	89.29	42.03	15.15	2.61
	63	Male	88.54	49.04	18.23	4.86
	62	Male	85.35	32.38	23.88	2.69
	61	Female	93.97	43.05	28.04	3.99
	60	Female	80.65	38.59	23.55	2.97
	60	Female	84.69	32.70	20.64	1.33
	60	Female	84.82	42.62	21.10	2.08
	60	Female	88.11	36.40	18.52	2.15
	60	Female	95.00	31.91	19.88	1.77
	60	Female	94.23	38.52	20.57	2.25
	60	Female	86.80	38.69	12.99	2.85
	60	Female	82.25	39.77	22.24	2.02
	60	Female	95.50	36.97	18.73	1.92
	60	Female	91.00	45.79	23.75	3.10
	60	Male	87.64	35.64	15.54	1.64
	60	Male	91.34	41.75	20.53	2.80
	60	Male	91.01	38.63	23.61	3.09
	60	Male	93.06	36.52	18.52	1.98
	60	Male	91.56	44.18	26.80	2.79
	60	Male	95.54	46.31	22.15	3.26
	60	Male	90.16	43.87	21.51	2.89
	60	Male	87.82	47.08	18.49	2.87
	60	Female	95.32	44.74	20.81	3.86
	59	Female	89.19	57.57	13.01	5.12
	57	Male	85.99	54.13	15.76	4.15
	57	Male	91.27	53.33	14.24	4.39
	57	Male	86.27	44.47	19.63	3.47
	56	Male	87.02	39.42	13.83	1.97
	54	Female	84.35	44.61	19.95	3.90
	50	Female	96.74	56.54	11.25	5.12
	49	Female	87.75	44.45	15.98	2.91
	46	Male	87.85	60.64	11.03	5.44
	46	Female	94.25	48.83	14.20	3.49
	46	Female	89.55	43.61	15.02	3.15
	41	Female	93.16	61.30	9.18	5.97
	36	Female	94.37	45.39	12.48	3.41
	30	Female	90.20	55.45	19.49	4.73
	28	Female	90.48	44.74	11.53	3.25
	24	Female	92.51	61.09	9.70	4.19
**Average**	57	40% Male	89.46	43.71	18.35	3.23

Supplementary table 1. Healthy control information on age and sex with total IgG1 fucosylation (Total fuc), galactosylation (Total gal), bisecting GlcNAc (Total bis) and sialylation (Total sial).

Sonneveld et al, supplementary table 4

Sex	Age	Hemo-lysis	C3d	Hb	Total Fuc	Total Gal	Total Bis	Total Sial	anti-RBC Fuc	anti-RBC Gal	anti-RBC Bis	anti-RBC Sial
Female	28	H			94.17	33.19	24.21	0.08	93.23	31.53	2.41	0.53
Female	30	N	0	13.9	89.00	58.00	15.00	6.00	81.00	21.00	15.00	6.00
Female	44	N	0	12.6	93.00	57.00	19.00	7.00	81.00	20.00	18.00	4.00
Male	80	N	0	14.7	97.00	35.00	12.00	3.00	97.00	15.00	21.00	2.00
Female	78	N	1	8.7	95.00	42.00	11.00	4.00	85.00	53.00	14.00	8.00
Male	68	U	1	6.3	93.75	51.07	9.87	7.38	93.46	46.18	8.26	7.78
Male	77	N	1	5.5	95.05	40.66	8.10	4.27	94.89	44.38	6.36	5.25
Female	30	N	1	7.7	85.00	44.42	21.34	3.54	96.54	20.85	21.20	2.41
Female	20	H	1	8.2	91.56	38.91	5.11	2.47	83.97	36.36	4.17	3.06
Female	48	N	0		89.94	46.21	15.87	4.70	66.85	23.70	18.96	8.74
Male	81	U	0		87.76	45.61	17.57	5.06	82.61	31.74	16.18	7.06
Female	54	N	1	14.5	91.76	29.64	16.81	1.65	94.60	28.22	20.65	3.12
Female	26	U	0		96.37	43.02	14.42	3.34	73.31	38.83	10.08	8.24
Male	74	U	0		90.69	30.30	11.78	1.63	33.22	50.05	32.67	19.00
Female	77	N	1	12.9	86.25	36.83	14.54	2.76	93.83	30.76	3.35	4.15
Female	84	H	1	6.9	94.63	34.17	4.70	2.58	91.16	22.39	5.48	5.93
Male	63	U	1	8.7	93.85	39.21	5.42	1.71	85.32	35.59	4.82	10.47
Male	69	N	0	8.4	91.09	37.15	17.36	1.88	88.57	55.74	3.26	5.76
Male	71	N	0	14.7	68.71	32.09	18.43	2.14	82.71	25.52	16.60	2.04
Male	74	U	0		94.42	36.70	10.88	2.22	87.56	31.43	17.92	3.92
Female	82	N	1	9.2	93.93	27.67	17.88	1.93	89.99	32.24	9.32	8.19
Male	74	U	0		93.53	33.08	11.89	2.03	88.76	31.41	12.56	5.24
Male	63	U	1	6.9	88.15	35.88	18.66	2.21	80.94	37.39	18.54	3.70
Female	64	U	0	7.3	95.42	29.60	20.01	1.38	94.28	32.17	14.87	4.25
Male	67	N	0	15.8	83.11	31.25	19.90	3.13	77.96	28.52	17.52	5.09
Male	66	U	1		88.83	38.28	14.60	1.85	77.11	31.91	9.65	1.72
Female	63	H	1		89.54	44.86	10.67	1.07	93.84	46.64	7.73	0.03
Female	78	H	1		92.70	37.26	8.38	3.49	95.05	28.90	3.59	1.35
Female	87	U	1	8.7	75.63	36.28	24.52	2.70	75.08	34.58	24.55	4.29
Female	44	U	1	8.2	88.83	38.28	14.60	1.85	91.30	28.33	17.54	3.93
Female	59	U	1	11.1	93.27	23.26	19.52	0.93	91.15	27.18	23.68	3.40
Female	76	H	1	7.7	81.73	47.10	9.59	4.00	64.00	59.98	7.97	9.24
Female	72	H	1	6.0	84.89	35.80	21.49	4.15	91.97	25.62	15.09	2.64
Female	61	U	1		80.56	30.20	18.60	1.42	84.08	29.72	11.16	3.09
Male	19	H	0	11.0	87.04	44.36	15.46	0.81	82.17	44.80	15.00	0.10
Male	71	H	0		79.89	36.09	14.31	5.47	86.86	28.85	10.91	2.85
Female	56	N	0		79.53	40.88	12.08	5.37	78.60	39.36	14.31	12.90
Male	62	N	0		91.82	22.15	7.46	1.87	89.32	26.96	10.82	4.77
Female	59	U	1		88.60	21.76	14.33	1.38	88.17	23.67	17.78	2.40
Male	68	U	0	8.9	88.98	26.10	16.16	3.58	94.23	19.78	7.71	1.09
Male	29	H	0		94.62	29.48	7.89	1.93	92.10	40.11	8.26	6.67
Female	86	U	0	6.4	89.53	18.35	16.01	2.42	82.69	20.96	9.26	4.46
Female	81	U	0	7.4	85.10	27.18	13.32	3.52	90.14	25.74	10.83	2.31
Female	41	N	0		76.47	44.84	12.55	7.54	75.95	47.06	18.99	15.43
Female	79	U	0		75.99	29.14	14.09	2.81	81.92	38.19	15.88	8.30
Male	63	N	1		94.02	31.73	9.56	1.73	94.30	33.10	11.70	4.51
Male	69	H	0	10.5	78.35	36.43	10.61	2.11	76.12	35.21	8.12	3.18
Male	56	U	0	7.6	96.98	34.00	47.04	0.10	95.75	30.15	14.62	0.34
Male	78	N	0		95.09	41.48	45.82	0.23	97.67	28.59	11.95	0.79
Female	56	N	1		94.96	45.99	29.33	0.13	98.00	35.43	15.74	0.30
Male	93	N	0		96.68	38.99	46.49	0.12	83.88	66.60	14.91	1.01
Male	18	N	0	6.9	91.97	31.79	35.38	0.32	85.49	45.21	11.13	2.48
Female	71	N	0		92.82	39.18	29.84	0.11	95.30	59.81	16.07	0.38
Male	67	N	0		96.03	26.69	23.21	0.19	89.05	41.50	12.12	0.18
Female	36	N	0		95.83	63.12	30.11	0.08	94.53	34.34	15.97	0.60
Female	84	N	1		97.66	39.31	46.13	2.37	89.57	45.80	17.00	1.08
Male	86	H	0	6.6	89.77	33.82	11.75	2.70	93.25	26.31	3.88	1.53
Female	76	H	0	10.5	94.66	26.99	19.99	1.51	97.32	15.24	7.65	1.36
Female	59	N	0		92.40	39.79	23.38	0.21	95.23	56.00	4.84	5.06
Female	34	N	1		88.31	56.98	32.56	3.92	86.69	45.35	17.83	2.01
Male	82	H	0	7.9	95.00	42.00	26.00	3.00	84.00	36.00	18.00	4.00

	Sex	Age	Hemo-lysis	C3d	Hb	Total Fuc	Total Gal	Total Bis	Total Sial	anti-RBC Fuc	anti-RBC Gal	anti-RBC Bis	anti-RBC Sial
	Female	64	H	0	6.4	91.54	32.70	13.45	1.76	96.53	5.12	2.85	0.18
	Male	49	H	0	9.5	94.37	32.29	43.37	0.38	91.06	28.12	11.09	0.31
	Male	28	H	0		90.45	29.48	35.84	0.23	89.52	37.67	20.34	0.33
	Female	42	H	1	11.3	85.30	37.39	19.07	3.13	85.93	33.61	21.60	2.39
	Female	33	N	0		92.07	58.58	42.26	0.54	97.48	29.62	15.36	0.76
	Female	47	H	1	8.1	81.79	27.27	15.20	2.38	93.24	22.99	4.68	1.29
	Male	80	H	1	5.3	85.88	44.69	17.51	4.07	91.34	26.62	17.24	2.79
	Male	77	H	1	6.8	89.76	35.87	26.50	0.03	87.55	50.47	11.34	0.85
	Female	55	H	1	6.8	94.01	37.41	37.18	0.23	92.89	30.47	9.49	0.19
	Female	57	N	1	8.7	93.24	48.75	19.08	0.09	89.43	50.77	20.01	0.45
	Female	28	N			95.09	41.48	45.82	0.23	97.67	28.59	11.95	0.79
	Male	80	N	1	13.2	95.21	21.44	22.26	1.31	94.43	20.10	14.00	1.49
	Male	45	H	1	7.7	94.49	46.79	42.36	0.19	98.65	17.61	13.64	0.01
	Female	29	N	1	12.4	92.31	40.17	7.99	2.73	95.84	28.71	11.57	1.89
	Male	82	H	0	6.4	81.43	33.82	23.71	4.07	84.96	30.72	22.58	1.63
	Female	80	U	1	8.7	90.86	26.20	21.69	1.32	88.09	22.19	9.32	2.84
	Male	82	H	0		88.27	41.96	10.41	1.03	95.76	17.35	5.56	0.20
	Female	78	H	0		91.03	36.59	23.07	3.58	97.39	35.73	7.82	3.42
	Male	73	H	0		82.65	37.94	20.75	3.40	96.08	31.52	4.13	2.46
	Female	49	H	1		93.33	40.81	31.17	0.15	95.35	48.99	12.78	0.11
	Female	59	H	1	4.4	85.97	41.34	14.60	5.97	99.05	12.74	9.60	0.35
	Male	76	U	1	11.9	80.48	31.27	13.43	3.53	87.85	23.92	13.39	3.49
	Male	67	U	1	12.9	91.20	32.96	9.08	2.92	78.01	43.47	17.39	11.00
	Female	28	U	1	11.8	95.00	54.00	23.00	6.00	81.00	42.00	16.00	5.00
	Female	67	U	1	14.5	93.00	46.00	16.00	4.00	94.00	25.00	25.00	3.00
	Female	67	H	1		95.04	47.13	15.21	3.81	94.91	37.67	7.85	5.48
	Male	68	H	1		91.45	28.95	18.20	1.21	97.81	6.33	2.21	0.26
	Male	66	H	1		96.94	21.89	14.01	1.48	96.48	10.09	6.75	1.38
**Ave**	45%	61				89.99	37.35	19.74	2.41	88.40	32.87	12.82	3.57

Supplementary table 2. Patient information of patients with autoantibodies against red blood cells.

Haemolysis: N = No haemolysis, U = unknown haemolysis, H = haemolysis
C3d:0 = C3d negative, 1 = C3d positive
Hb = Hemoglobin level (g/dL)
Fuc = fucosylation, Gal = galactosylation, Bisec = bisection, Sial = sialylation
Ave = Average; average age for No haemolysis = 58, Unknown haemolysis = 66, Haemolysis = 61

Sonneveld et al, supplementary figure 1

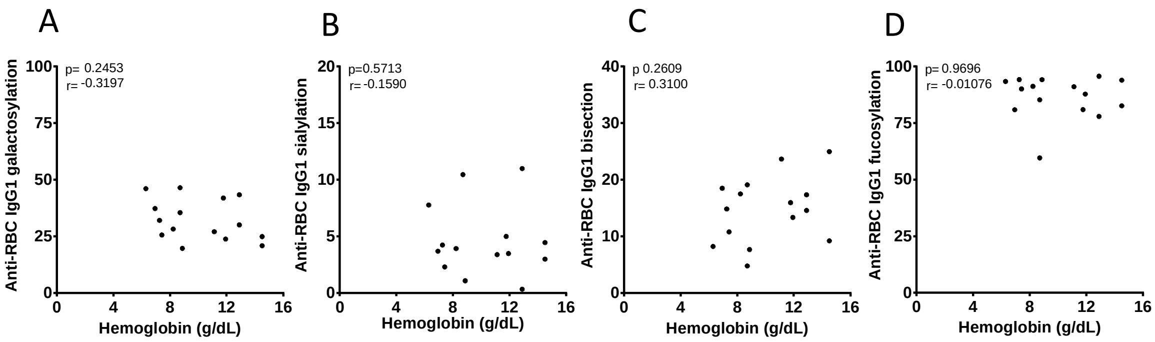

Supplementary figure 1 No correlation between anti-RBC specific glycosylation and severity of anemia in patients with unknown hemolysis. Anti-RBC IgG1 galactosylation, sialylation, bisecting GlcNAc, fucosylation (y-axis) and hemoglobin level (x-axis) for IgG1 is shown for (A-D) patients with unknown hemolysis. There is no correlation between anti-RBC IgG1 glycosylation and Hb level in patients with unknown hemolysis (A-D). Statistical analysis was done using the Pearson correlation test, after 5% FDR correction *p*-values ≤0.0074 were considered statistically significant.

Sonneveld et al, supplementary figure 2

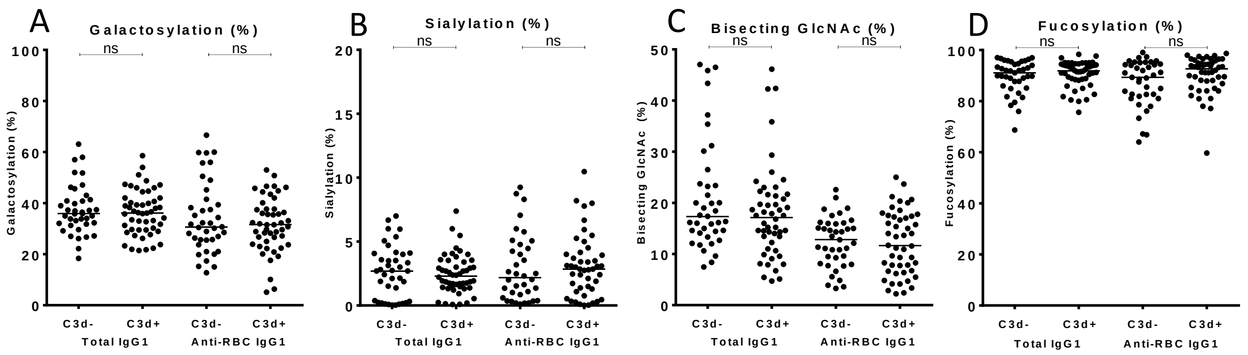

Supplementary figure 2 Fc-glycosylation does not influence complement-opsonization of RBC isolated from AIHA patients. The level of IgG1 glycosylation (y-axis) of either total- and anti-RBC specific IgG1 was not different from patients with C3d+ or C3d- RBC, as seen for galactosylation (A), sialylation (B), bisecting GlcNAc (C) or fucosylation (D). The data present individual observations with median. Statistical analysis was done using unpaired t-tests, after 5% FDR correction *p*-values ≤0.0074 were considered statistically significant.

Sonneveld et al, supplementary figure 3

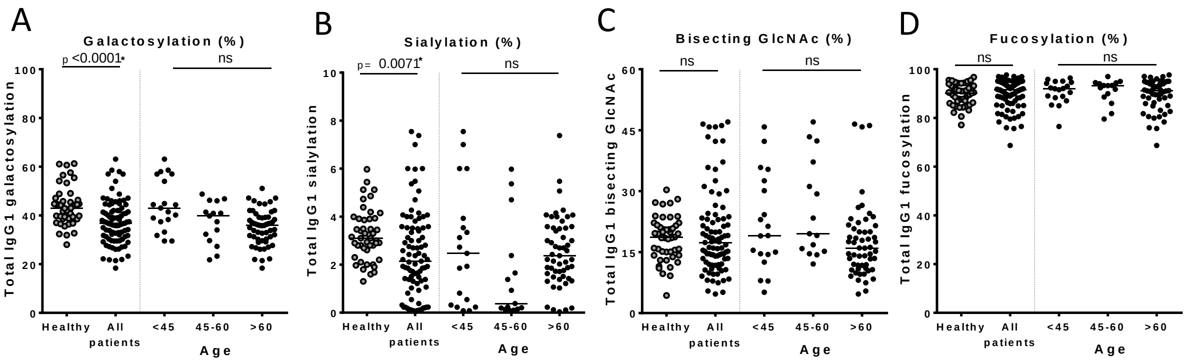

Supplementary figure 3. Lack of IgG1 galactosylation and sialylation in patients compared to healthy controls is not explained by age. Total IgG1 glycosylation (y-axis) in patients with autoantibodies against RBCs (black dots) is different compared to healthy controls (grey dots) (x-axis). Fc-galactosylation (A) and sialylation (B) are lower in patients with RBC autoantibodies. Fc-bisecting GlcNAc (C) and Fc-fucosylation (D) is comparable in both groups. No difference was found between patients in different age groups (x-axis) for total galactosylation (A), sialylation (B), bisecting GlcNAc (C) and fucosylation (D). The data present individual observations with median. Statistical analysis was done using a unpaired t-test (healthy vs. control) and Kruskall Wallis test (age groups), after 5% FDR correction *p*-values ≤0.0074 were considered statistically significant.
